# Pharmacological effects, molecular mechanisms, and pharmacokinetics of benzoylaconine: a systematic review

**DOI:** 10.3389/fphar.2025.1571153

**Published:** 2025-08-05

**Authors:** Huamei Zhuang, Hong Yao

**Affiliations:** ^1^ School of Pharmacy and Medical technology, Putian University, Putian, China; ^2^ Key Laboratory of Pharmaceutical Analysis and Laboratory Medicine, Putian University, Putian, China; ^3^ Department of Pharmaceutical Analysis, School of Pharmacy, Fujian Medical University, Fuzhou, China; ^4^ Fujian Key Laboratory of Drug Target Discovery and Structural and Functional Research, Fujian Medical University, Fuzhou, China

**Keywords:** benzoylaconine, toxicity, pharmacokinetics, pharmacological activity, molecular mechanisms

## Abstract

Benzoylaconine (BAC), a key active metabolite in traditional Chinese medicine, is derived from the subsoil roots of Fuzi (*Aconitum carmichaelii* Debx [Ranunculaceae, *Aconitum carmichaelii* Debx roots]). BAC has garnered considerable research attention because of its therapeutic effects against cardiovascular disease, inflammation, and arthritis, and this has led to continual updates in the literature. This systematic review summarizes evidence on the pharmacological effects, molecular mechanisms, and pharmacokinetics of BAC. PubMed and Web of Science were searched for relevant articles published between January 2000 and November 2024. Genes, proteins, and pathways related to the activity and therapeutic effects of BAC were identified. BAC usually targets proteins such as ACE2, IL-6, MAPK, PI3K, Akt, STAT3, TNF-α, and VEGF. The identified genes and proteins were subjected to protein–protein interaction analysis, molecular docking between BAC and protein hubs, and bioinformatic analyses (gene ontology, Kyoto Encyclopedia of Genes and Genomes, and disease ontology analyses). Protein–protein interaction analysis and molecular docking indicated IL-6, Akt1, and STAT3 as key targets of BAC. These findings offer theoretical insights into the potential therapeutic mechanisms of BAC and may inform its future development as a pharmacological agent.

## 1 Introduction

**FIGURE 1:**
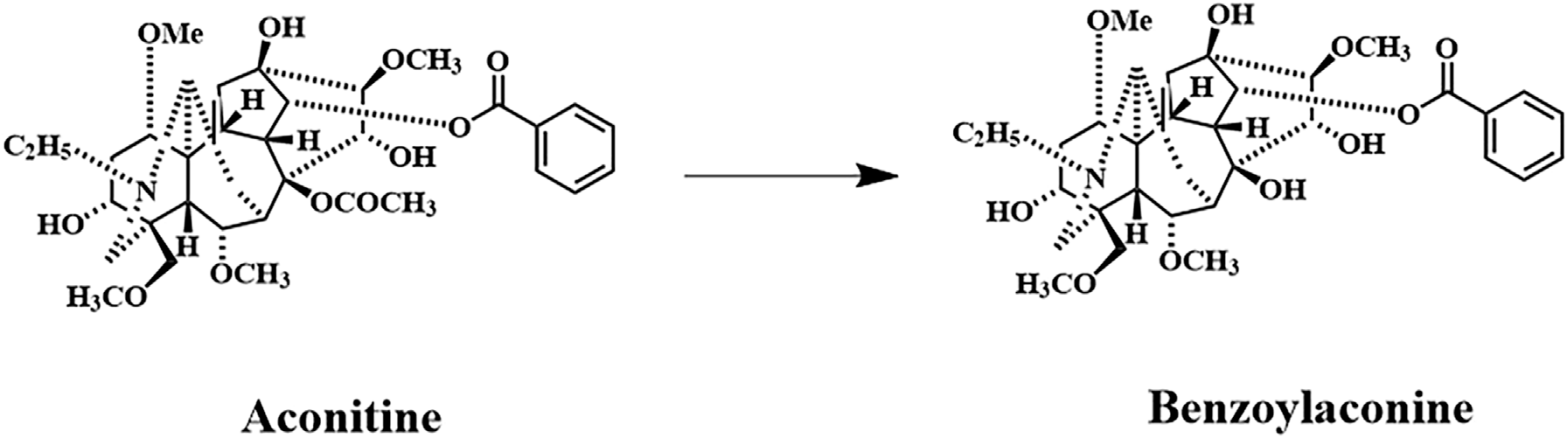
Primary metabolic pathway involving AC and BAC. Benzoylaconine; Chemical Abstracts Service registry number: 466–24-0; molecular formula: C32H45NO10; molecular weight: 603.7 g/mol.

Benzoylaconine (BAC) is a white crystalline compound that is soluble in organic solvents such as methanol, ethanol, isopropanol, and chloroform and slightly soluble in water. The oral bioavailability (OB) of BAC is 12.8, indicating limited absorption through the oral route. Nonetheless, BAC has a relatively high drug-likeness value of 0.25. BAC, a common monoester diterpenoid alkaloid (MDA), is a major active metabolite in the traditional Chinese medicine (TCM) Fuzi. Fuzi is widely used in TCM formulations such as Shenfu-Tang, Fuzi-Tang, Zhenwu-Tang, and Sini-Tang for cardiac support, diuresis, vasodilation, circulatory enhancement, and central analgesia. BAC is the hydrolysis product of aconitine (AC) (He et al., 2022) (Figure 1), which is derived during the processing of *Aconitum carmichaelii*. (He et al., 2022). The toxicity of BAC is approximately 100 times lower than that of AC. (Zhang et al., 2016; Ye et al., 2012). BAC has proven effective in mitigating arthritis, inflammation, cardiac injury, and psoriasis. (Li et al., 2021; Li et al., 2023; Yu et al., 2020; Zhang et al., 2024).

The present systematic review summarizes evidence on the pharmacological effects, molecular mechanisms, and pharmacokinetics of BAC. This study was conducted adhering to standard methods: inclusion and exclusion criteria were predefined to ensure reproducibility, comprehensive search strategies were used across multiple databases (PubMed and Web of Science), and structured data extraction and quality assessment were performed in accordance with the Preferred Reporting Items for Systematic reviews and Meta-Analyses (PRISMA) guidelines. The use of these standard methods differentiates the present study from narrative reviews, which typically offer a broader thematic overview without a systematic search or quality appraisal. Our ultimate goal was to provide valuable insights for future translational research.

## 2 Methods

**FIGURE 2:**
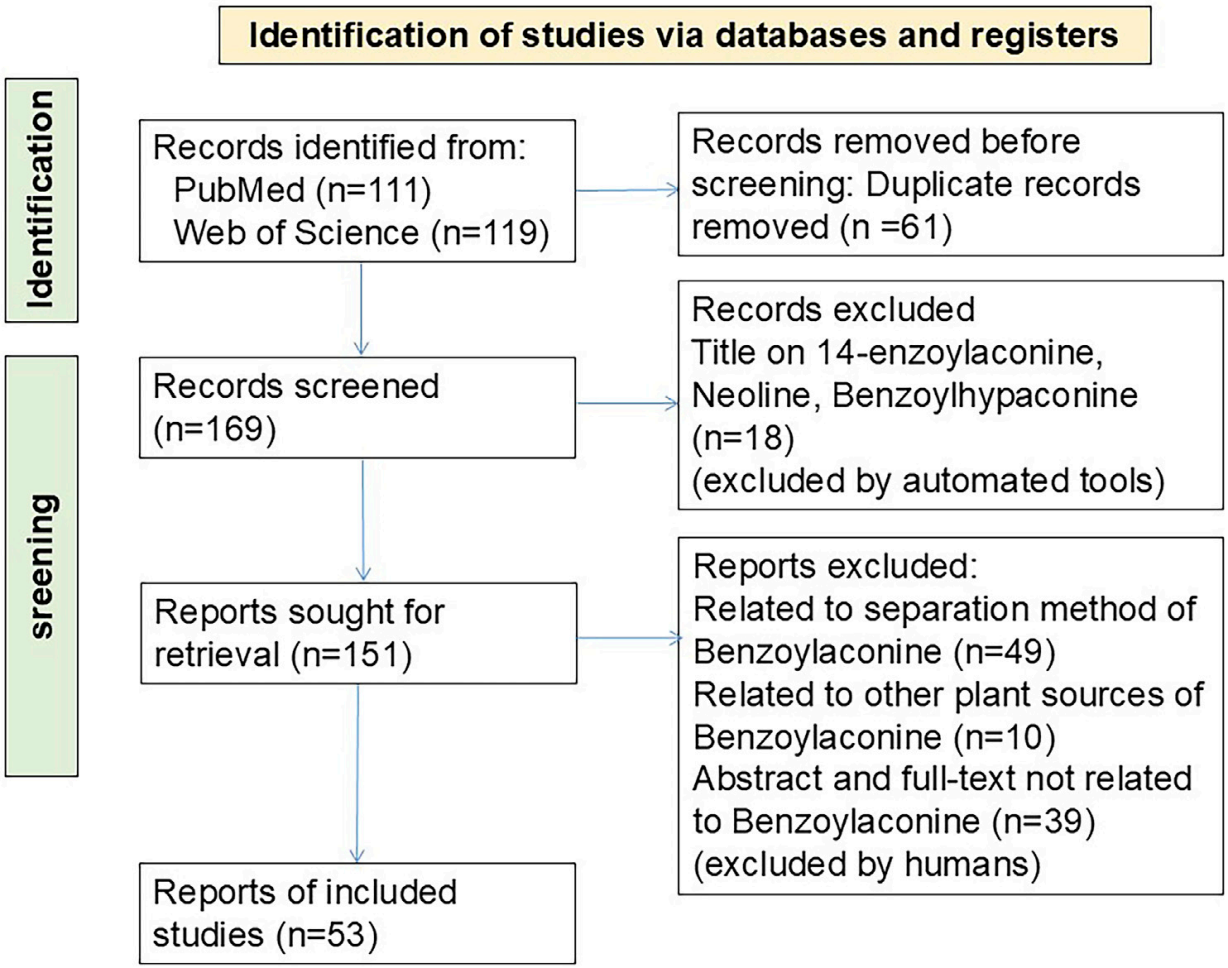
PRISMA flowchart depicting article selection.

PubMed and Web of Science were systematically searched for BAC-related studies published between January 2000 and November 2024. The article language was restricted to English. The search strategy was as follows: “(Benzoylaconine) AND (“2000/01/01” [Date - Publication]: “2024/11/30” [Date - Publication])” and “(ALL=(Benzoylaconine)) and DOP=(20000101/20241130).” A total of 111 records were retrieved from PubMed and 119 from Web of Science. Duplicate articles (n = 56) were removed using Zotero Literature Manager (https://www.zotero.org/). Articles unrelated to BAC were manually excluded by reviewing titles and abstracts. Moreover, studies focusing on the synthesis and development of BAC formulations were excluded. The final analysis included 53 articles. A flowchart depicting the article selection process (Figure 2) was created by following the PRISMA guidelines. (Page et al., 2021). The study protocol was registered with the International Prospective Register of Systematic Reviews database (identifier: CRD420250639795).

In this comprehensive review, we explored the pharmacokinetics, toxicity, and pharmacological effects of BAC. In addition, through protein–protein interaction (PPI) analysis, we investigated the genes and proteins that mediate BAC’s therapeutic effects against various diseases. PPI revealed multiple high-node-degree or hub proteins for molecular docking with BAC. We further performed bioinformatic analyses, which involved gene ontology (GO), Kyoto Encyclopedia of Genes and Genomes (KEGG), and disease ontology (DO) analyses. Our findings may inform future research on BAC and have implications for the safety and clinical application of BAC-containing TCMs.

## 3 Pharmacokinetics

### 3.1 Pharmacokinetic profiles

Pharmacokinetic studies provide valuable insights into *in vivo* drug metabolism and efficacy, toxicity reduction, and clinical application. High-performance liquid chromatography–mass spectrometry can be used to simultaneously determine the *in vivo* concentration–time profiles of several *Aconitum* alkaloids, such as BAC, AC, mesaconitine (MA), and hypaconitine (HA). (Fan et al., 2021). Fan et al. reported that BAC had a peak residence time of 60.75–69.59 min and an average residence time of 284.57–292.56 min in rats, indicating slow clearance. (Fan et al., 2021). High dosages consistently increased the apparent volume of distribution/OB ratio of *Aconitum* alkaloids, thereby reducing the OB of each alkaloid. (Fan et al., 2021).

Ye et al. intravenously injected rats with a mixture of eight alkaloids (AC, MA, HA, and BAC) and their parent compounds and eventually found these alkaloids in the rats’ blood. (Ye et al., 2012). Therefore, the parent compounds, which were more toxic than were the corresponding secondary metabolites, were rapidly eliminated from the body. Zhang et al. demonstrated that the maximum plasma concentrations of BAC, AC, and aconine (ACN) were reached within 1 h of the injection, indicating the rapid absorption of these alkaloids into circulation. (Zhang et al., 2016). Notably, the clearance rate and absorption efficiency were higher for AC than for BAC and ACN. AC primarily accumulates in muscles, whereas BAC and ACN primarily accumulate in the heart and kidneys. The bloodstream does not easily absorb BAC, and BAC is consequently excreted mainly through feces.

Yougui-Wan, a TCM preparation used for treating osteoporosis due to kidney yang deficiency, contains Fuzi. (Wang et al., 2023). The rates of BAC absorption and clearance in rats with osteoporosis due to kidney yang deficiency were higher and lower, respectively, than in those with osteoporosis due to kidney yin deficiency. Yougui-Yin, another TCM preparation for treating osteoporosis, contains six types of *Aconitum* alkaloids, among which BAC has the lowest clearance rate and longest residence time. (Zhang J. et al., 2019). Pharmacokinetic studies involving Shenfu-Tang revealed that although the ginsenoside Rg1 markedly enhanced BAC absorption and accelerated AC metabolism, it exerted nonsignificant effects on ACN. (Xu et al., 2020). BAC is the primary active metabolite in Sini-Tang, which is used to treat myocardial infarction (MI) and heart failure. (Zhou et al., 2019). A study highlighted a lower level of systemic BAC exposure and a lower rate of drug clearance in MI rats than in control rats. A Phase I clinical trial analyzed the pharmacokinetics of BAC after intravenous injection of Shenfu-Tang powder in 18 healthy volunteers. (Zhang et al., 2008). The pharmacokinetic half-life of BAC was relatively short (<1 h). Furthermore, the plasma concentration of BAC peaked at 30 min for moderate doses and 45 min for high and low doses.

Combining Fuzi with other TCM preparations can reduce its toxicity and improve therapeutic efficacy. (Zhang M. et al., 2019). A pharmacokinetic study underscored the therapeutic potential of BAC in rats orally treated with Fuzi plus Dahuang Fuzi decoction. (Liu X. et al., 2014). Notably, Ganjiang (Zingiber officinale Roscoe [Zingiberaceae; Zingiberis Rhizoma]) may promote AC clearance and enhance BAC absorption when coadministered with Fuzi. (Peng et al., 2013). Due to the reduced toxicity of Chuanwu (Aconitum carmichaelii Debx. [Ranunculaceae; Aconiti Radix]) when used in combination with honey, Chuanwu has been used in combination with honey for a long time. Wu et al. reported that a combination of Chuanwu and honey resulted in a higher peak plasma concentration, a higher area under the plasma concentration–time curve, a shorter time to reach the peak plasma concentration, and a longer half-life of clearance for BAC than did Chuanwu alone. (Wu et al., 2022). The detoxifying and synergistic effects of honey may be attributable to its ability to reduce the toxicity of diester diterpenoid alkaloids (DDAs) while increasing the *in vivo* bioactivity of MDAs. In their pharmacokinetic study on BAC in rats, Zhi et al. elucidated the mechanisms underlying the detoxifying effect of Chaihu on Caowu, supporting the rationale for using steaming and boiling methods in Caowu-based treatments. (Zhi et al., 2020).

The pharmacokinetic properties of drugs are influenced by protein binding. Zhou et al. performed multispectroscopic analysis, molecular docking, and dynamic simulation to investigate the interaction between human serum albumin and BAC. (Zhou et al., 2023). Molecular docking indicated that the electronic domains of BAC’s nitrogen and benzene ring skeletons were essential for complex formation. In silico simulation revealed stability changes and key residues involved in the binding of AC analogs with human serum albumin—for example, TRP-214, LEU-219, and LEU-238.

The key features of pharmacokinetic studies on BAC are summarized in Table 1. Further animal and human studies are required to analyze the pharmacokinetic properties of BAC when administered alone.

**TABLE 1:** Pharmacokinetic studies on BAC.

Species	Research objects	Dose (mg/kg)	Pharmacokinetic parameters				Reference
			AUC_(0-t)_ (ng/mL·h)	T_max_ (h)	C_max_ (ng/mL)	T_1/2_ (h)	
Administration route:Gavage							
SD rats	BAC	0.5	121.4 ± 45.77	1.16 ± 0.59	34.51 ± 21.62	5.479 ± 2.276	Fan et al. (2021)
		1	280.6 ± 107.6	0.841 ± 0.327	122.8 ± 62.49	4.508 ± 1.424	
		2	987.7 ± 382.9	1.013 ± 0.392	359.9 ± 89.75	4.985 ± 1.471	
SD rats	CW	92.34 ± 0.49	698.3 ± 52.41	0.75 ± 0	1.54 ± 0.09	6.057 ± 0.163	Wu et al. (2022)
	CW–honey	90.65 ± 5.96	1239.8 ± 41.13	0.25 ± 0	12.26 ± 0.73	8.309 ± 0.148	
SD rats	BAC	20	215 ± 38.1	0.333 ± 0.105	35.2 ± 11.5	11.0 ± 2.85	Xu et al. (2020)
	BAC + Rg1	20 + 20	318 ± 72.5	0.333 ± 0.00	63.1 ± 11.1	10.3 ± 4.21	
SD rats	CW	0.001	6.74 ± 0.68	0.5 ± 0.00	4.44 ± 0.72	1.12 ± 0.18	Zhi et al. (2020)
	Hezi-CW		2.97 ± 0.60	0.83 ± 0.29	1.79 ± 0.25	0.58 ± 0.17	
SD rats	SND	1.895 mg/mL	40.44 ± 13.61	0.71 ± 0.13	12.82 ± 5.80	12.38 ± 4.02	Zhou et al. (2019)
MI SD rats			10.79 ± 7.53	1.44 ± 0.17	3.56 ± 2.10	15.49 ± 2.99	
Wistar rats	Fuzi	10 mL/kg	8.2 ± 2.7	0.63 ± 0.14	3.4 ± 0.6	3.69 ± 0.25	Song et al. (2015)
	Fuzi-Mahuang		12.2 ± 2.3	0.67 ± 0.20	3.1 ± 0.3	4.36 ± 1.13	
SD rats	Fuzi	0.45 g/mL	5.928 ± 0.324	0.586 ± 0.098	1.16 ± 0.05	3.11 ± 0.235	Peng et al., 2013)
	Fuzi–Ganjiang		6.958 ± 0.392	0.365 ± 0.149	2.08 ± 0.16	3.154 ± 0.155	
Administration route: Oral							
SD rats	CW	0.1963	32.45 ± 9.17	0.19 ± 0.04	7.887 ± 4.192	13.82 ± 3.10	Liu J. et al. (2014)
		0.2067	40.43 ± 13.04	4.17 ± 0.75	4.813 ± 3.923	9.9 ± 1.8	
SD rats	Fuzi	0.2813	486.9 ± 255.5	0.6 ± 0.3	151.6 ± 129.3	9.4 ± 2.3	Liu X. et al. (2014)
	DFD	0.2778	144.6 ± 36.8	0.2 ± 0.2	17.2 ± 10.7	28.7 ± 12.5	
SD rats	Shen-Fu prescription	0.93	23.34 ± 13.01	0.29 ± 0.17	6.56 ± 3.32	1.10 ± 0.68	Ouyang et al. (2018)
Kidney yang deficiency SD rats	YGW	1764	8.87 ± 3.94	0.17 ± 0.00	13.25 ± 1.38	5.08 ± 1.29	Wang et al. (2023)
Kidney yin deficiency SD rats			2.65 ± 0.35	0.17 ± 0.00	6.64 ± 0.87	0.40 ± 0.12	
SD rats	BAC	1	13.54 ± 2.29	0.31 ± 0.17	3.99 ± 1.20	9.49 ± 0.49	Zhang et al. (2016)
SD rats	YGY	15 mL/kg	271.3 ± 39.4	8.4 ± 5.6	18.3 ± 3.8	10.7 ± 3.6	Zhang J. et al. (2019)
Administration route:Intravenous injection							
healthy volunteers	Shen-Fu powder	1.1035	14.12 ± 3.31	0.75 ± 0	9.120 ± 2.02	0.739 ± 0.031	Zhang et al. (2008)
		1.4677	21.87 ± 1.31	0.5 ± 0	11.80 + 0.290	1.036 ± 0.047	
		1.8428	25.47 ± 0.54	0.75 ± 0	17.58 + 1.76	0.8 ± 0.23	

Abbreviations: SD, sprague dawley; AUC_0–t_, area under the plasma concentration–time curve from 0 to the last quantifiable time point; C_max_, peak plasma concentration; T_max_, time to reach C_max_; T_1/2_, half-life of clearance; CW (Caowu), Aconiti Kusnezoffii Radix; Fuzi, Aconiti Lateralis Radix Praeparata; DFD, Dahuang Fuzi decoction (composed of Radix et Rhizoma Rhei, Fuzi, and Radix et Rhizoma Asari); YGW, You-Gui-Wan; YGY, You-Gui-Yin; Hezi-CW, caowu processed with chebulae fructus; SND, sini decoction; MI, myocardial infarction; Mahuang, Ephedrae Herba; Ganjiang, Rhizoma Zingiberis.

### 3.2 Effects of BAC on efflux transporters

Efflux transporters—such as P-glycoprotein (P-gp), breast cancer resistance protein (BCRP), and multidrug resistance–associated protein 2 (MRP2)—are key regulators of drug pharmacokinetics and herb–herb or herb–drug interactions. (Ye et al., 2013). P-gp and BCRP primarily mediate the transport of AC, whereas MRP2 mediates that of BAC. (Dai et al., 2015). A study demonstrated that *Aconitum* alkaloids upregulated P-gp expression in LS174T and Caco-2 cells; the order of the effect magnitude was as follows: AC > BAC > ACN. (Wu et al., 2016). Moreover, AC and BAC increased the transport activity of P-gp. Intracellular BAC can increase adenosine triphosphate (ATP) levels and mitochondrial mass. Furthermore, BAC considerably upregulates the expression of MRP2 and BCRP and increases the efflux activity of MRP2 by activating the Nrf2-mediated pathway. (Wu et al., 2018). Therefore, BAC may serve as a quality indicator for *Aconitum*-derived botanical drugs.

## 4 Toxicity

*Aconitum* alkaloids have acute and high toxicity, which induces severe arrhythmia that can result in death. AC, MA, and HA are the main and highly toxic alkaloids in the genus *Aconitum*. (Ji et al., 2019; Ye et al., 2019). Accidental ingestion of *Aconitum* can be fatal. (Dickens et al., 1994). Although *Aconitum* roots are highly toxic, (Ito et al., 1996), they have for centuries been used in traditional medicine across East Asia. According to the 2020 Chinese Pharmacopoeia, only processed *Aconitum* roots are permitted for clinical use in Fuzi preparations. *Aconitum* alkaloids are metabolized primarily by esterases. AC is hydrolyzed at the C-8 position to form BAC and at both the C-8 and C-14 positions to form ACN (Figure 1). (Mizugaki et al., 1998; Wada et al., 2005).

Pharmacological experiments in Sprague Dawley rats indicated that 0.01 mg/kg AC and 10 mg/kg ACN improved cardiac function, whereas 2 mg/kg BAC impaired it. (Liu et al., 2017). DDAs are 100–700 times more toxic than are MDAs. During aconite processing, *Aconitum* alkaloids are converted from DDAs into MDAs and then into alkylamine diterpenoid alkaloids, thereby reducing toxicity. (Bisset, 1981). However, caution should be exercised to avoid overprocessing and overhydrolysis of BAC into ACN. (Li et al., 2016). A study measuring BAC and similar alkaloids in rapidly dried and fresh-cut *Aconitum* revealed that although traditional processing reduces toxicity, it leads to >85.2% alkaloid loss. (Zhang D. K. et al., 2017).

Ephedrae Herba (Mahuang) (Ephedra sinica Stapf [Ephedraceae; Ephedrae Herba)–Fuzi is a traditional formula used to treat the common cold, asthma, and rheumatoid arthritis (RA). A study reported that the combination of *Aconitum* and Ephedra poses a risk of *Aconitum* alkaloid poisoning, as evidenced by the widespread distribution of nine alkaloids, including BAC, in the heart, liver, spleen, lungs, kidneys, and brain of treated individuals. (Ren et al., 2017). Prolonged use of this formula may lead to drug accumulation. Therefore, patients using formulations that contain ephedrine and *Aconitum* alkaloids should be closely monitored to prevent adverse effects on the cardiovascular and central nervous systems. (Song et al., 2015). Oral administration of AC induced bradycardia and hypotension in rats, consistent with AC poisoning in humans. (Zhang P. et al., 2017). These findings indicate that the metabolites of AC and BAC have antihypertensive properties.

## 5 Pharmacological effects

### 5.1 Cardioprotective effects

Fuzi is widely used for treating heart failure and related cardiac diseases. BAC, a major active metabolite in Fuzi, holds promise for the prevention and treatment of cardiovascular diseases, inflammation, arthritis, and other conditions (Table 2).

**TABLE 2:** Molecular mechanisms underlying the pharmacological effects of BAC.

Cell/Animal	Model	BAC dose	Effect	Molecular mechanisms	References
Cardiovascular system protection					
Wistar Rat	MI	Gavaged, 0.0068 mg/kg	Promoting cardiac function, alleviating myocardial hypoxia, inhibiting inflammatory response fibrosis in heart tissue	↓VEGF, ↓PKM2, ↓GLUT-1, ↓LDHA, ↓TNF-α, ↓IL-1β, ↓IL-6, ↓COX2	Xing et al. (2022)
C57BL/6 mice, ACE2−/− mice	HF	Oral, 3, 10, 30 mg/kg	Enhancing cardiac function in heart failure	ACE2, ↓p38, ↓ERK, Mitochondrial, ↓ROS, ↓NF-κB	Zhang et al. (2024)
SD rats	IR	20 mg/kg	Alleviated myocardial injury	↑AMPK, ↑PGC-1	Chen Z. et al. (2022)
H9c2 cells		50, 75 µM	improves mitochondrial function	↑ROS, ↑LDH	
Anti-inflammatory activity and Antirheumatic activities					
SD rats	OA	Gavaged, FZD, 0.2114 ± 0.028 mg/kg	Anti-OA, restored cartilage degeneration, ameliorating pain behavior, benefitting cartilage anabolism, increased cell viability and wound healing capacity, recovering histopathological alterations	↑Col2, ↓MMP13, ↓Col10, ↓PI3K, ↓Akt	Chen L. et al. (2022)
SD rats	CIA	Gavaged, 1.5 mg/kg	Alleviate the degree of swelling, arthritis index and pathological lesions of the sacroiliac gland	↓PGE2, ↓IL-1β, ↓TNF-α, ↓VEGF, ↓IgG, ↓STAT1, ↓STAT3	Li et al. (2022)
KM mice	inflammatory ear, paw edema	intravenous injection, 10 mg/kg	Anti-inflammatory for RA therapy	↓TNF-α, ↓IL-1β, ↓NF-κB, ↓p65	Gai et al. (2020)
RAW264.7 cells		5, 18, 40 μg/mL	reduce the viability of activated macrophages		
Wistar rats	AIA	Gavage, 0.126, 0.252, 0.504 mg/kg	Anti-inflammatory, inhibiting immune organs (spleen and thymus), attenuating paw swelling, infiltration of inflammatory cells and synovial hyperplasia	↓IL-1β, ↓IL-17A, ↓COX-2	Li et al. (2021)
SW982 cell		0, 5.10 µM	Anti-inflammatory	↓IL-6, ↓MAPK, ↓Akt, ↓IκB-α, ↓p65, ↓IL-8	Yu et al. (2020)
SD rats and KM mice	OA	Orally, 536.6 ± 6.16, 813.1 ± 3.5 mg/kg	Against OA, attenuated joint pain, prevented articular degeneration, suppress chondrocyte hypertrophy and extracellular matrix degradation	↓Col10, ↓Mmp2, ↓Sox5, ↓Adamts4/5/9, ↑Col2	Zhang et al. (2020)
HFLS-RA fibroblast-like synoviocytes	RA	1,000 μg/mL	Anti-inflammatory and anti-rheumatic activities	↓PGE2, ↓IL-6, ↓IL-1β, ↓TNF-α, ↓TLR4, ↓HIF-1α, ↓VEGFA	Zhang et al. (2021)
SD rats	SH	0.6, 2, 6; 3, 10, 30 mg/kg	Antihypertensive effects, enhancing vasodilation and alleviating vascular inflammation	↑Akt/eNOS, ↑NO, ↑Ang (1–7), ACE2; ↓TNF-α, ↓IL6, ↓COX-2, ↓ACE, ↓AngII, ↓IKB-α	Zhang et al. (2022)
HUVECs cell		25, 50, 100 μM			
RAW264.7 macrophage cells		0.1, 1, 10, 100, 500 μM	Anti-inflammatory	↓IL-6, ↓TNF-α, ↓IL-1β, ↓ROS, ↓NO, ↓PGE2, ↓iNOS, ↓COX-2, ↓NF-κB, ↓IκBα, ↓p65, ↓TLR4, ↓TAK1, ↑JNK, ↑p38, ↑ERK	Zhou et al. (2021)
Others					
HepG2 cells		25, 50, 75 µM	Induces mitochondrial biogenesis, increased mitochondrial mass	↑mtDNA copy number, ↑cellular ATP, ↑OXPHOS, ↑AMPK	Deng et al. (2019)
Balb/c mice		10 mg/kg			
SD rats			Mitochondrial abnormalities	↑mitochondrial energy metabolism	Zhang D. K. et al. (2017)
HaCaT keratinocytes	Psoriasis	10, 20, 40 µM	Anti-psoriasis, inhibiting cell proliferation, the release of inflammatory factors, and the accumulation of Th17 cells	↓STAT3	Li et al. (2023)
C57BL/6 mice		1, 3 mg/kg			
C57BL/6J mice	cholestatic mouse	Oral, YCZFD, 2.383 ± 0.103, 4.765 ± 0.205 μg/kg	Against CLF, decreased liver injury, and fibrosis biochemical indicators	↓PDGFRβ, ↓PI3K-Akt	Meng et al. (2024)
Wistar rats and KM mice		350 μg/cm^2^	analgesic and anti-inflammatory effects	↑surface tension, ↑skin permeation, ↑interaction strength	Liu et al. (2019)
SD rats	IR	intraperitoneal injections, 5,10,20 mg/kg	against skeletal muscle I/R injury	↑IF1, ↑AMPK, ↑Nrf2, ↑HO-1	Cui et al. (2024)
C2C12 cells		60 μM			

Abbreviations: SD, sprague dawley; MI, myocardial infarction; HF, heart failure; I/R, ischemia/reperfusion; AIA, adjuvant-induced arthritis; LDH, lactate dehydrogenase; OA, osteoarthritis; FZD, fuzi decoction; CIA, collagen-induced arthritis; KM, kunming; RA, rheumatoid arthritis; SH, spontaneous hypertension; YCZFD, yinchenzhufu decoction.

BAC considerably reduces the serum levels of superoxide dismutase, MDA, creatine kinase–myocardial band, cardiac troponin T, and cardiac troponin I in MI rats and downregulates the expression of hypoxia- and inflammation-related genes such as *VEGF*, *PKM2*, *GLUT-1*, *LDHA*, *TNF-*α, *IL-1*β, *IL-6*, and *COX2*. (Xing et al., 2022). Furthermore, BAC markedly improves cardiac function, reduces infarct size, inhibits inflammatory cell infiltration, and mitigates myocardial fibrosis.

BAC was demonstrated to inhibit angiotensin II-induced cellular hypertrophy and fibrosis in primary cardiomyocytes and fibroblasts from rats and mitigate cardiac dysfunction and remodeling in mice with thoracic aortic constriction. (Zhang et al., 2024). Regarding heart failure treatment, BAC directly targets angiotensin-converting enzyme 2 (ACE2), thereby inhibiting the activation of the p38/extracellular signal-regulated kinase signaling–mediated mitochondrial reactive oxygen species (ROS) and nuclear factor (NF)-κB pathways. BAC appears to be a promising ACE2 agonist and therapeutic agent for heart failure because it can regulate mitochondrial ROS release and inflammatory activation, thereby improving cardiac function. (Zhang et al., 2024). An *in vitro* study indicated that BAC enhanced the survival rate of H9c2 cells subjected to oxygen–glucose deprivation and reperfusion. (Chen L. et al., 2022). Furthermore, BAC upregulates the expression of phosphorylated adenosine monophosphate–activated protein kinase (AMPK) and peroxisome proliferator-activated receptor gamma coactivator 1α. It also improves mitochondrial function, reduces oxidative stress and apoptosis, and mitigates myocardial injury *in vivo*.

**FIGURE 3:**
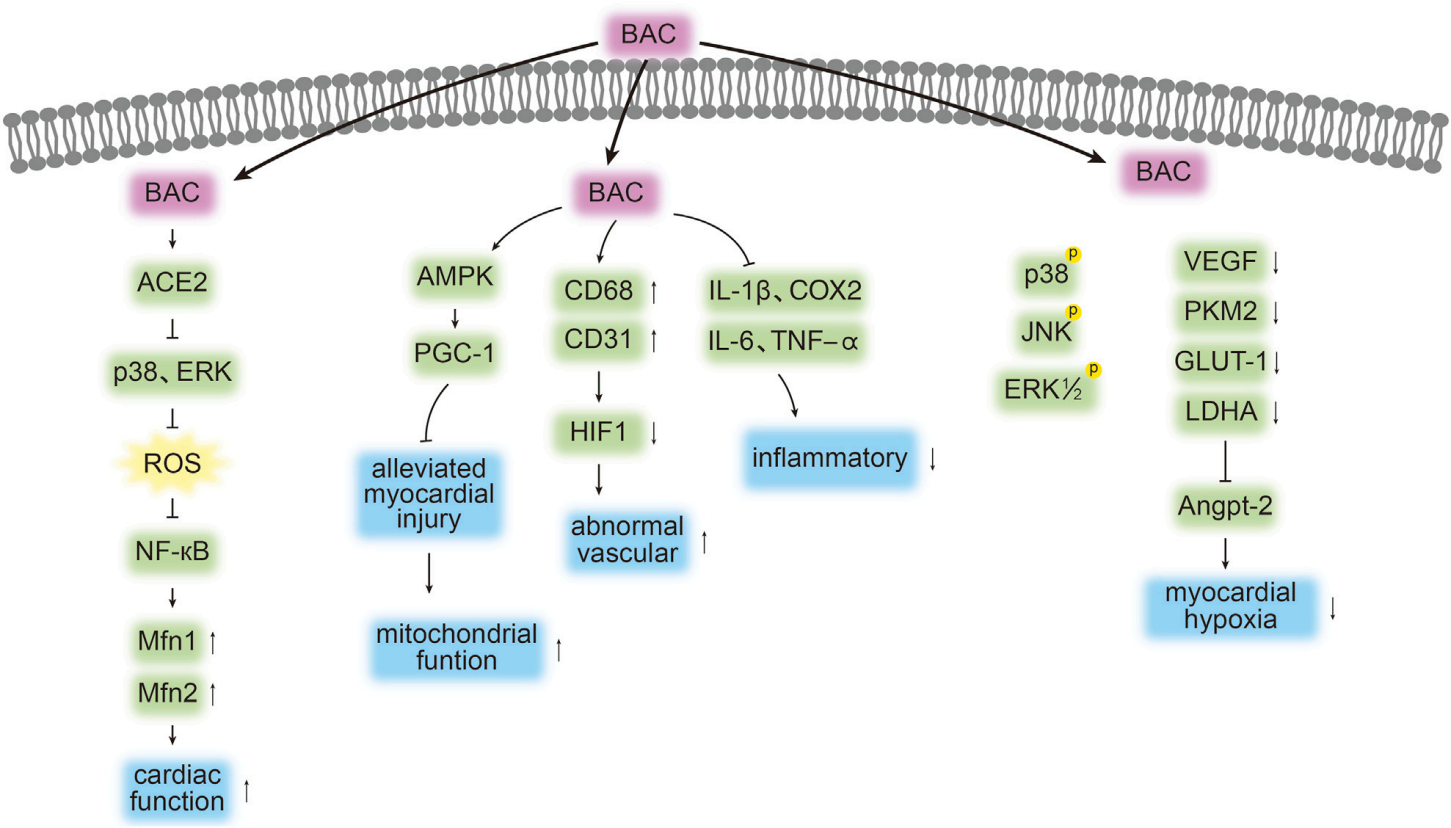
Targets and pathways associated with the cardioprotective effects of BAC. The symbols “↓”, “↑”, “┤,” and p denote protein downregulation, upregulation, inhibition, and phosphorylation, respectively. Arrows (“→”) indicate signal transduction or promotion.

The mechanisms through which BAC treats cardiovascular disease, as outlined in the literature, are depicted in Figure 3 and Table 2. Nonetheless, further studies are required to comprehensively identify the targets of BAC and the pathways mediating its cardioprotective effects.

### 5.2 Anti-inflammatory and antiarthritic effects

BAC exhibits strong anti-inflammatory activity in lipopolysaccharide (LPS)-stimulated macrophages. It was demonstrated to reduce primary and secondary paw swelling in rats with adjuvant-induced arthritis and to mitigate collagen-induced arthritis. (Gu et al., 2018). In addition, BAC markedly alleviated joint tissue inflammation, prevented bone destruction, and reduced serum interleukin (IL)-1β and IL-17A levels. It also downregulated the expression of cyclooxygenase (COX)-1 and COX-2 in synovial tissues. (Li et al., 2021). BAC further inhibited IL-1β-induced gene and protein expression of IL-6 and IL-8 in human synovial SW982 cells. Moreover, BAC suppressed the activation of mitogen-activated protein kinase (MAPK) and protein kinase B (Akt), inhibited the degradation of inhibitor of κB-α, and prevented the phosphorylation and nuclear translocation of p65. (Yu et al., 2020). One study reported that BAC strongly inhibited the proliferation of human-fibroblast-like synoviocytes derived from adult RA synovial tissues, (Zhang et al., 2021), highlighting the *in vitro* antirheumatic activity of this alkaloid. This activity may be mediated through downregulation of inflammatory cytokines, such as prostaglandin (PGE)2, IL-6, IL-1β, and tumor necrosis factor (TNF)-α; hypoxia-inducible factor (HIF)-1α; vascular endothelial growth factor (VEGF); and Toll-like receptor (TLR)4 expression.

The aforementioned findings are consistent with those reported by another study, in which BAC considerably inhibited the release of proinflammatory cytokines and mediators, such as IL-6, IL-1β, TNF-α, ROS, nitric oxide, and PGE2. (Zhou et al., 2021). BAC also dose-dependently blocked LPS-induced increases in the protein levels of inducible nitric oxide synthase and COX-2. Moreover, it suppressed the phosphorylation and degradation of inhibitor of κB-α and the nuclear translocation of p65, thereby inhibiting LPS-induced NF-κB activation. In addition, BAC blocked LPS-induced increases in the levels of phosphorylated c-Jun N-terminal kinase, p38, and extracellular signal-regulated kinase. It further inhibited LPS-induced phosphorylation of transforming-growth-factor-β-activated kinase one in activated RAW264.7 macrophages. (Zhou et al., 2021). Considering BAC’s anti-inflammatory properties, Gai et al. developed a drug delivery system by encapsulating BAC in nanoparticles to regulate inflammatory responses. (Gai et al., 2020). Activated macrophages treated using this system exhibited 70% and 66% lower TNF-α and IL-1β levels, respectively, compared with corresponding levels in the control without BAC nanoparticles. Liu et al. reported that the use of penetration enhancers in BAC-loaded transdermal patches intensified the analgesic and anti-inflammatory effects of BAC, supporting its potential for treating inflammatory pain. (Liu et al., 2019).

RA is a chronic, systemic autoimmune disease of the joints. BAC has been demonstrated to substantially reduce swelling and arthritis index scores in rats with collagen-induced arthritis and downregulate IL-1β, VEGF, PGE2, TNF-α, and immunoglobulin G by inhibiting the Janus kinase/STAT pathway. In a rat model of osteoarthritis, BAC downregulated aberrant expression of Col10, Mmp2, Sox5, and Adamts4/5/9 and upregulated that of Col2 in cartilage. (Zhang et al., 2020). *In vitro* experiments using rat cells revealed that treatment with BAC-containing serum considerably promoted chondrocyte proliferation and regulated *Col2*, *Mmp1*, *Adamts9*, and *Aggrecan* expression. These findings highlight the molecular mechanisms underlying BAC-mediated inhibition of chondrocyte hypertrophy and extracellular matrix degradation. Thus, BAC serves as an analgesic and a regulatory agent. In a study on osteoarthritis, Fuzi-Tang was found to involve similar molecular mechanisms. (Chen Z. et al., 2022). BAC, the main active metabolite in Fuzi-Tang, improved pain-related parameters, mitigated histopathological changes, promoted cartilage anabolism (by upregulating Col2 expression), and inhibited cartilage catabolism (by downregulating matrix metalloproteinase 13 and Col10 expression), thereby reversing cartilage degeneration in rats with osteoarthritis. Experiments and network pharmacology analyses have indicated that the phosphoinositide 3-kinase/Akt pathway mediates the antiarthritic effect of BAC. Notably, BAC may be the active metabolite responsible for the anti-inflammatory and immunosuppressive effects of Mahuang–Fuzi–Xixin-Tang. (Tang et al., 2015).

**FIGURE 4:**
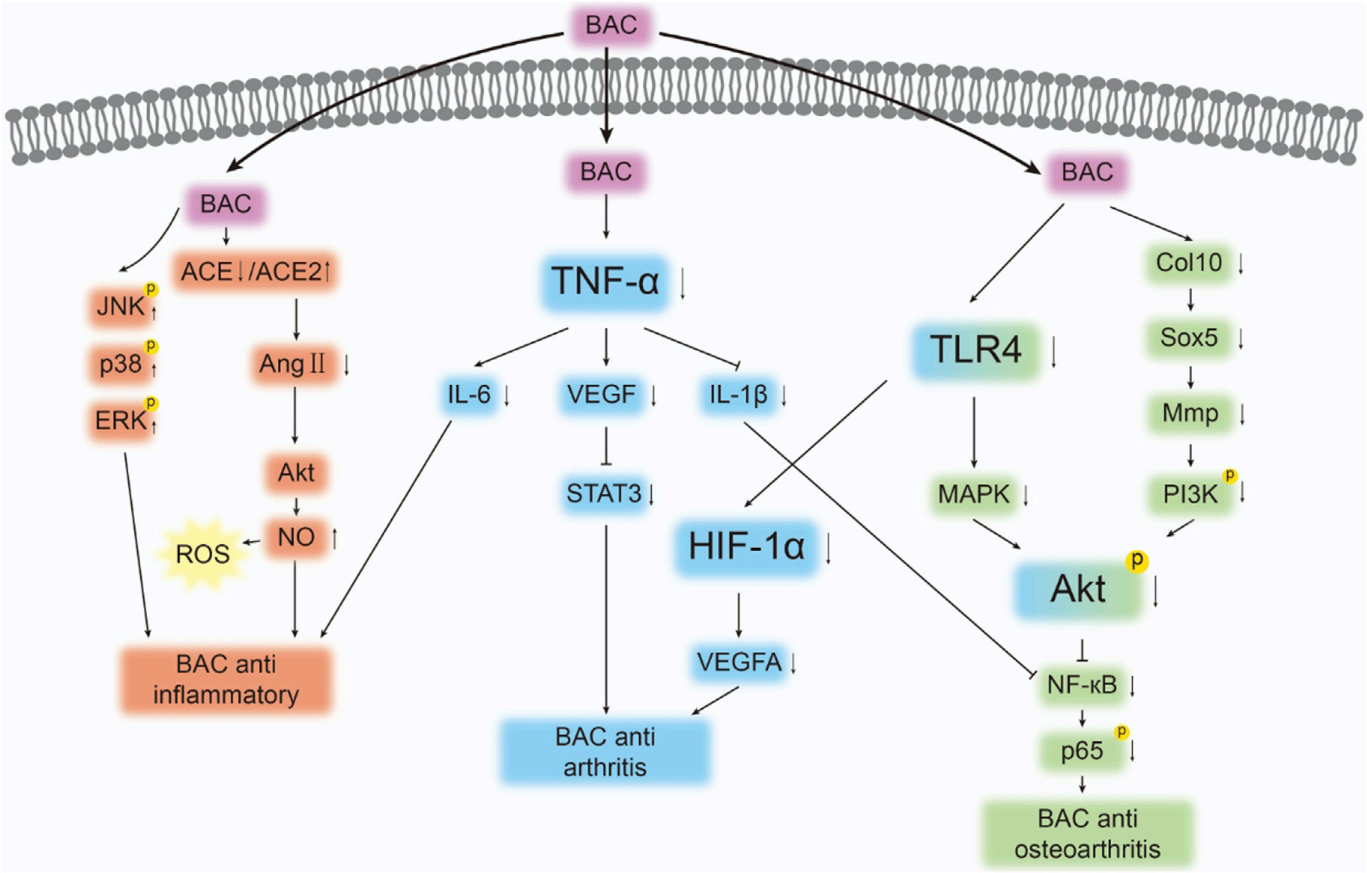
Targets and pathways associated with the anti-inflammatory and antirheumatic effects of BAC. The symbols “↓”, “↑”, “&boxvl;,” and p denote protein downregulation, upregulation, inhibition, and phosphorylation, respectively. Arrows (“→”) indicate signal transduction or promotion.

The primary targets of BAC and the pathways that mediate its activity against inflammation and RA are depicted in Figure 4 and Table 2. Further studies are required to comprehensively explore BAC targets in inflammation and RA as well as associated therapeutic pathways.

### 5.3 Others

BAC modulates mitochondrial energy metabolism and exerts antipsoriatic and anticholestatic effects against liver fibrosis.

In HepG2 cells, BAC dose-dependently increased the mass of the mitochondria, copy number of mitochondrial DNA, cytosolic level of ATP, and expression of proteins involved in oxidative phosphorylation. (Deng et al., 2019). Moreover, this alkaloid dose-dependently upregulated the expression of proteins involved in the AMPK pathway both *in vivo* and *in vitro*. In HepG2 cells, BAC increased cell viability without influencing cell proliferation. *In vitro* data suggest that BAC increases the rate of oxygen consumption in mice and activates AMPK signaling in the heart, liver, and muscles. Notably, the ester bond at the C-8 position, hydroxyl group at the C-3 position, and ethyl group on the nitrogen atom in BAC substantially contribute to its effects on mitochondrial energy metabolism. (Zhang D. K. et al., 2017). These findings highlight the therapeutic potential of BAC against diseases involving mitochondrial dysfunction.

Psoriasis is a common polygenic skin condition characterized by inflammatory infiltrates, keratinocyte hyperproliferation, and immune cell accumulation. BAC may ameliorate psoriasis symptoms by inhibiting keratinocyte proliferation, inflammatory factor release, and Th17 cell accumulation. (Li et al., 2023). In TNF-α/LPS-stimulated HaCaT keratinocytes, BAC markedly reduced the protein and mRNA levels of inflammatory cytokines by inhibiting STAT3 phosphorylation. Therefore, BAC may slow psoriasis progression and thus serve as a promising therapeutic agent for psoriasis.

Yinchenzhufu decoction (YCZFD) is a TCM preparation with hepatoprotective effects. YCZFD contains seven primary metabolites, including BAC, which can considerably reduce serum metabolite levels, liver injury, and fibrosis index scores in mice with cholestatic liver fibrosis (CLF). (Meng et al., 2024). This study revealed that the expression of platelet-derived growth factor receptor-β (PDGFRβ) was upregulated in the liver of mice with CLF. (Meng et al., 2024). However, YCZFD treatment downregulated the expression of PDGFRβ. The protective effect of BAC against CLF is mediated primarily through regulation of the PDGFRβ/phosphoinositide 3-kinase/Akt pathway.

BAC has been demonstrated to protect skeletal muscle tissue from ischemia/reperfusion injury; increase cell viability; elevate the superoxide dismutase level; and reduce creatine kinase, lactate dehydrogenase, ROS, MDA, and proapoptotic factor levels both *in vivo* and *in vitro*. (Cui et al., 2024). Mechanistically, BAC upregulates ATPase inhibitory factor 1 expression, promotes AMPK phosphorylation, facilitates Nrf2 nuclear translocation, and induces heme oxygenase-1 expression.

## 6 Network pharmacology analysis

**FIGURE 5:**
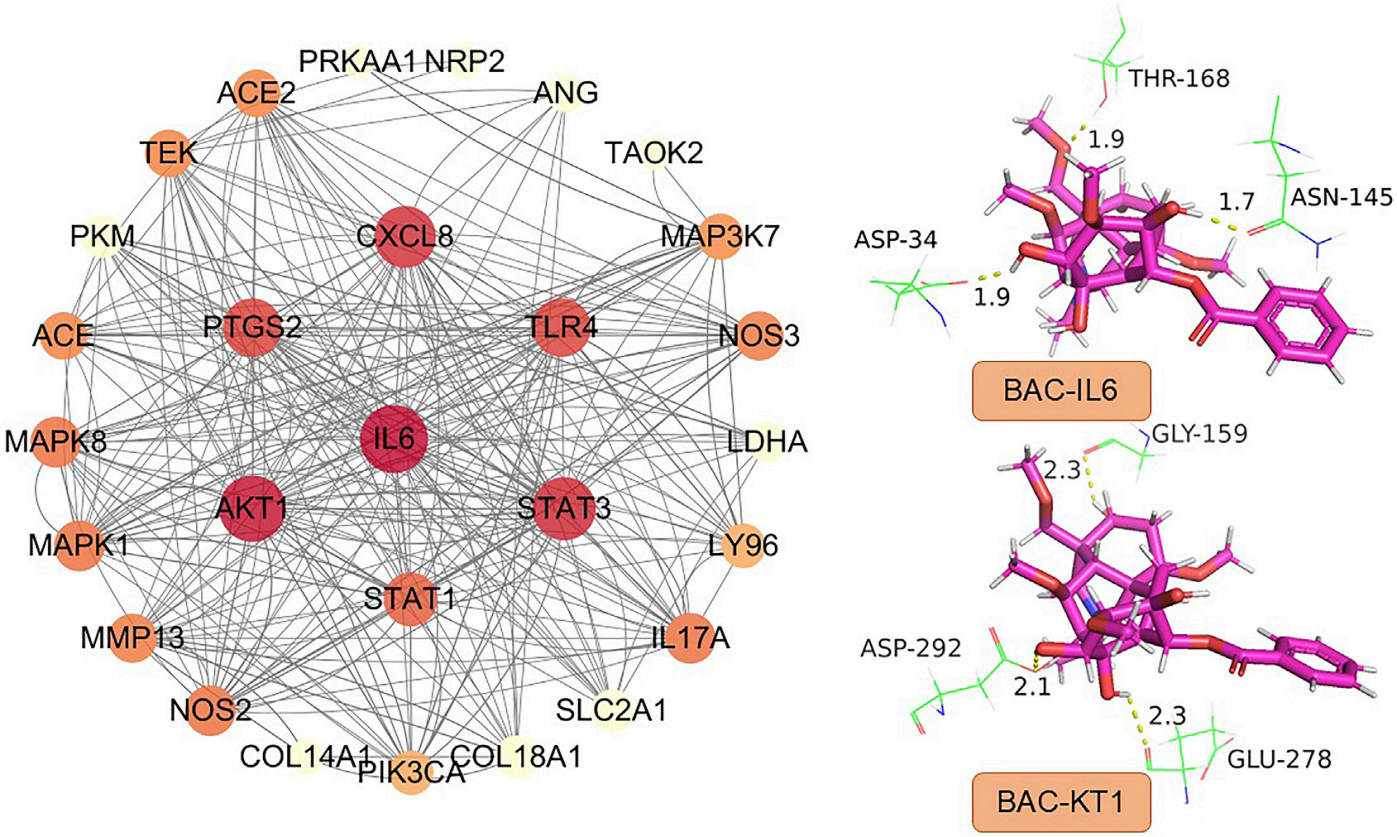
PPI network constructed using BAC targets and high-node-degree proteins.

To investigate the potential relationships of BAC with its target proteins and genes, we performed PPI network analysis by using a previously reported method (Xie et al., 2020). The resulting network contained 31 nodes and 146 edges, excluding hidden standalone nodes. IL-6, Akt1, and STAT3 emerged as the primary proteins with high connectivity in the PPI graph (Figure 5).

Molecular docking performed using SYBYL-X (version 1.3; Tripos Inc., St. Louis, MO, United States) revealed three key amino acid residues involved in the binding of IL6 with BAC: Asp, Thr, and Asn. These residues formed three intramolecular hydrogen bonds, each of length <2 Å: 1.9 Å for Asp34, 1.9 Å for Thr168, and 1.7 Å for Asn145. The amino acid residues involved in the binding of Akt1 with BAC were Asp, Gly, and Glu. The corresponding hydrogen bonds had length smaller than 2.5 Å; the lengths were 2.1 Å for Asp292, 2.3 Å for Gly159; and 2.3 Å for Glu278, indicating strong binding affinity (Figure 5). The score for molecular docking between IL-6 and BAC was 6.04, whereas that for molecular docking between Akt1 and BAC was 7.53. To simplify the simulation, explicit water molecules were excluded from the docking system. Our findings indicate that BAC strongly interacts with IL-6 and Akt1, highlighting the need for further in-depth mechanistic studies. IL-6 and Akt1 appear to play key roles in the PPI network of BAC, as indicated by their high degrees and docking affinities. A study demonstrated that, under hypoxic conditions, IL-6 promoted apoptosis and inhibited autophagy in cardiac stem cells by suppressing the phosphorylation of STAT3, suggesting the existence of a regulatory axis. (Li et al., 2025). Regarding systemic inflammation, cannabidiol mitigates cardiovascular injury by downregulating IL-6, STAT3, and HIF-1α and upregulating endothelial nitric oxide synthase; these findings implicate the IL-6/STAT3 pathway in the regulation of oxidative stress and inflammation. (Tepebasi et al., 2024). In pulmonary hypertension, the IL-6/gp130 pathway in CD4^+^ T cells drives pathogenesis through STAT3 phosphorylation and Th17 cell activation. Notably, IL-6 knockdown or gp130 deficiency can ameliorate pulmonary hypertension. (Ishibashi et al., 2024). In RA, the IL-6/STAT3 pathway serves as an “autoimmune adaptive axis,” enabling immune cells and synovial fibroblasts to perpetuate inflammation and resist treatment through epigenetic and noncoding RNA–mediated mechanisms. (Kumar and Mangla, 2025). Together, the findings indicate that the IL-6/STAT3 pathway mediates apoptosis, inflammation, and immune regulation in cardiovascular disease and arthritis. (Qin et al., 2025; Abdelaziz et al., 2025). Thus, this pathway holds promise as a therapeutic target for BAC.

After constructing the PPI network, we performed gene enrichment analyses—GO, KEGG, and DO analyses—with the identified proteins and genes. These analyses were performed using relevant bioinformatic tools (http://www.bioinformatics.com.cn/). (Sherman et al., 2022)

**FIGURE 6:**
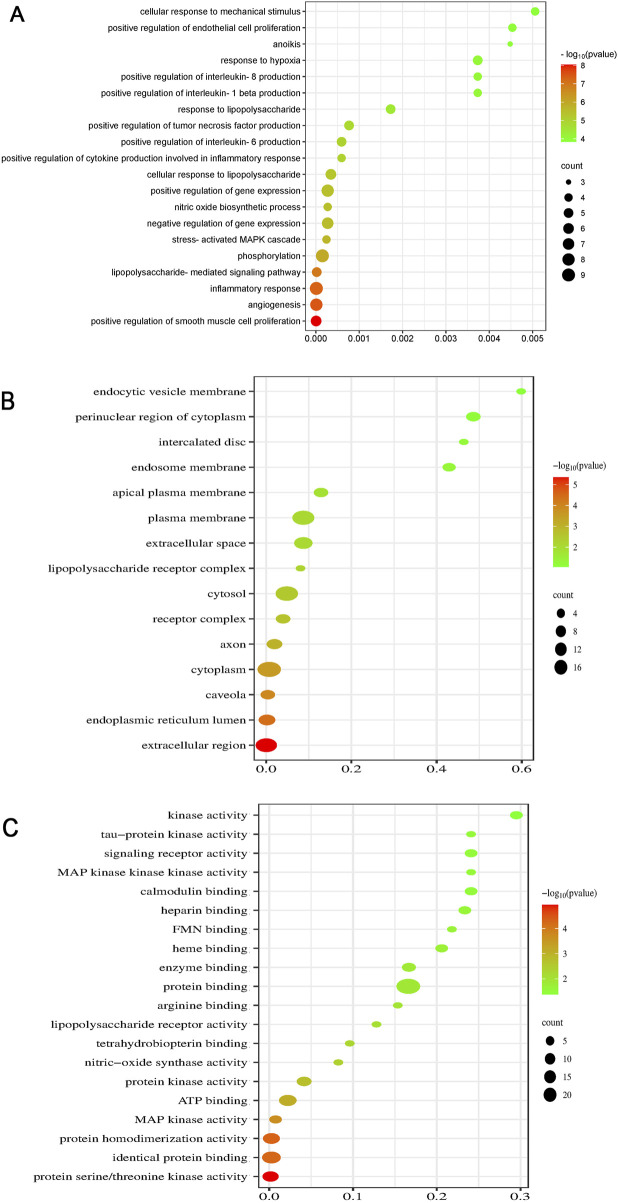
Dot plots for GO analysis: **(A)** BP, **(B)** CC, and **(C)** MF analyses.

GO is used to determine the properties of genes and gene products. A biological analysis revealed that the key processes influenced by BAC include inflammation, angiogenesis, LPS-mediated signaling, positive regulation of smooth muscle cell proliferation, positive regulation of IL-8 production, positive regulation of IL-1β production, and phosphorylation of the stress-activated MAPK cascade (Figure 6A), supporting our conclusions. A cellular component analysis indicated localization in the extracellular region, endoplasmic reticulum lumen, cytoplasm, caveolae, cytosol, LPS receptor complex, plasma membrane, and extracellular space (Figure 6B). A molecular function analysis suggested that the function of BAC primarily includes protein serine/threonine kinase activity, identical protein binding, protein homodimerization, MAP kinase activity, and ATP binding (Figure 6C).

**FIGURE 7:**
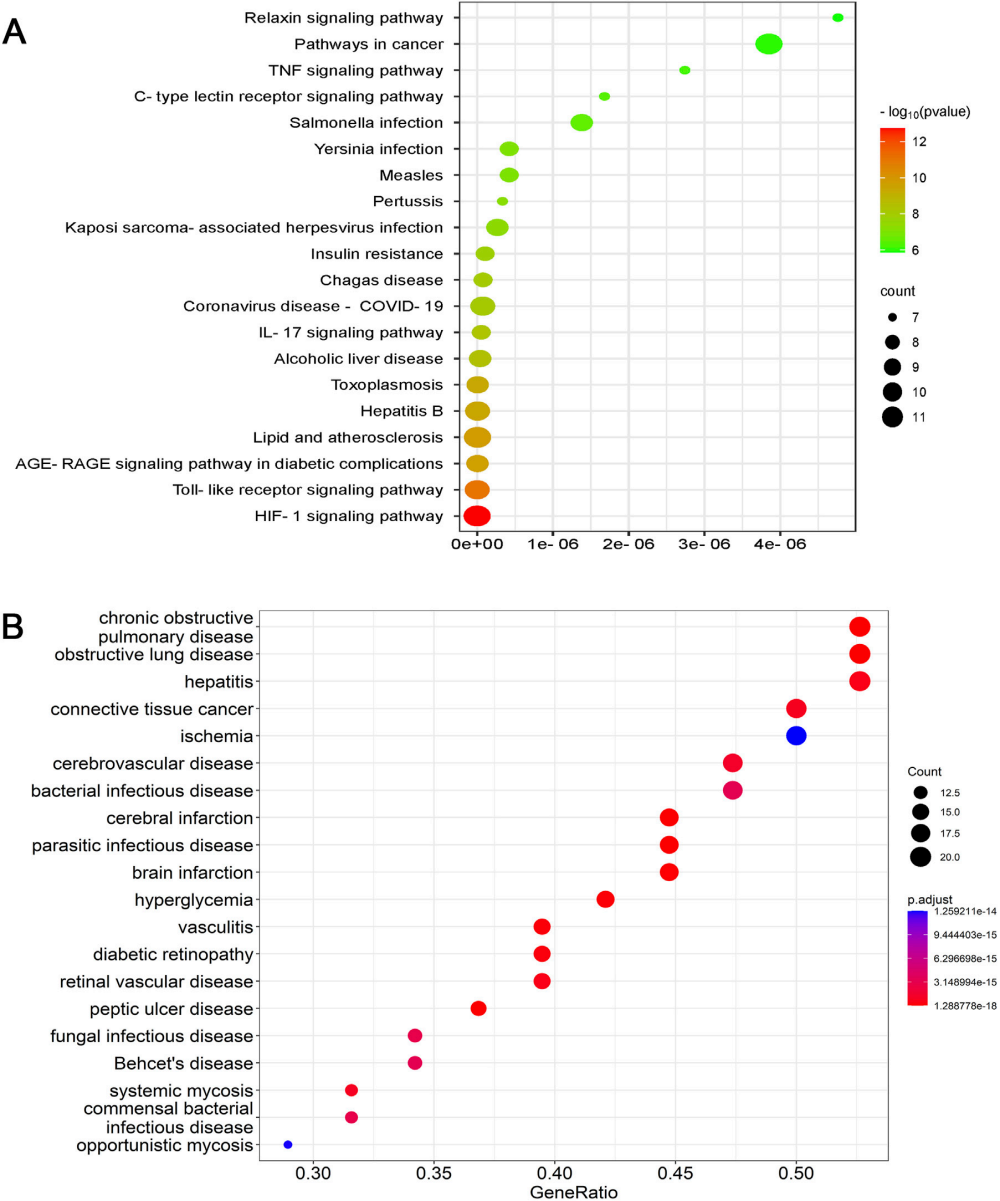
Dot plots for **(A)** KEGG and **(B)** DO analyses.

The KEGG database contains information on known intermolecular interactions, such as biochemical and metabolic reactions. Figure 7A presents the major pathways potentially influenced by BAC—for example, HIF-1 signaling, cancer development, TLR signaling, advanced glycation end product–receptor for advanced glycation end product signaling (in diabetes-related complications), lipid metabolism and atherosclerosis development, and hepatitis B development. The predicted pathways, particularly those involving cancer and HIF-1α signaling, should be validated through Western blotting and reverse transcription quantitative polymerase chain reaction to elucidate the precise mechanisms underlying the therapeutic effects of BAC.

DO is a key analytical tool used in studies concerning the genetic basis of pathogenesis. In the present study, DO analysis indicated that genes and proteins associated with BAC were also associated with chronic obstructive pulmonary disease, obstructive pulmonary disease, hepatitis, connective tissue cancer, cerebrovascular disease, bacterial infectious diseases, cerebral infarction, parasitic infections, brain infarction, and ischemia (Figure 7B). Although BAC is known to alleviate inflammation and reduce cardiocerebrovascular risk, very few studies have investigated its effects against chronic obstructive pulmonary disease, connective tissue cancer, bacterial infections, and myocardial ischemia, indicating a need for further investigation.

On the basis of the literature, we hypothesize that BAC exhibits immunomodulatory activity in infectious diseases. Some studies have underscored the vital role of TLR4–NF-κB signaling in parasitic infections. For instance, in poultry birds with *Heterakis gallinarum* infection, elevated TLR4 level was associated with elevated proinflammatory cytokine (e.g., IL-1β and interferon-γ) levels and tissue damage. (El-Saied et al., 2024). Similarly, *Plasmodium falciparum* elicited a TLR-mediated proinflammatory response, upregulating TNF-α and downregulating IL-10. (McCall et al., 2007). This mechanism may be analogous to the immunomodulatory effect of BAC. In viral hepatitis (hepatitis B or C), dysregulated microbiota and cytokine profiles (e.g., IL-6 and TNF-α) exacerbate liver pathology, (Padilha et al., 2024), highlighting the need to regulate the crosstalk between TLR and NF-κB, as demonstrated in *Echinococcus granulosus* infection. (Taha et al., 2025). Parasitic nematodes such as *Trichinella spiralis* reduce the levels of proinflammatory cytokines (e.g., IL-6 and TNF-α) to ameliorate metabolic disorders. (Kang and Yu, 2024). BAC may mimic this immunoregulatory mechanism.

## 7 Conclusion and implications

We systematically reviewed BAC-related studies published between January 2000 and November 2024. This review focused on the therapeutic effects of BAC against cardiovascular and cerebrovascular diseases, inflammation, arthritis, and related conditions. Our results indicate that BAC regulates multiple signaling cascades, exerting diverse biological effects on various targets.

BAC enhances cardiac function, alleviates myocardial hypoxia, and inhibits inflammatory response–induced fibrosis in cardiac tissue. It also exerts antiarthritic effects by restoring degenerated cartilage, alleviating joint pain, reversing histopathological changes, promoting cartilage anabolism, inhibiting cartilage catabolism, and improving chondrocyte viability and wound healing capacity. Moreover, BAC reduces sacroiliac gland swelling, foot and plantar swelling, arthritis index scores, and the extent of pathological lesions (e.g., inflammatory cell infiltration and synovial hyperplasia). It further suppresses immune organs (e.g., the spleen and thymus) and inhibits splenocyte proliferation, exerting strong anti-inflammatory effects.

Although promising findings have been obtained, several limitations persist in BAC-related research. (1) Animal Models versus human diseases: Although animal models are indispensable in preclinical research, interspecies differences in genetics, metabolism, and disease pathophysiology limit their translational relevance. For example, murine models often fail to fully replicate human immune responses and complex comorbidities. (2) Effect of low OB: Low OB (attributable to factors such as first-pass metabolism or poor intestinal absorption) can reduce systemic exposure and thus drug efficacy. In such cases, high dosing (which carries a high risk of toxicity) or alternative delivery methods (e.g., nanocarriers and prodrugs) should be considered to achieve therapeutic concentrations. (3) Delivery route trade-offs: Oral administration offers convenience at the cost of variable absorption; it is thus suitable for chronic conditions. Intravenous delivery, suitable for acute conditions, ensures full bioavailability but is invasive. Subcutaneous or intramuscular routes strike a balance between bioavailability and patient compliance, particularly for biologics. Thus, the delivery route should be tailored to drug characteristics and clinical indications to optimize treatment efficacy and adherence. However, few studies have compared the various routes of BAC monotherapy delivery. Further research in this area is warranted.

Owing to its multitarget mechanism and low toxicity, BAC offers therapeutic advantages over conventional anti-inflammatory and cardiovascular drugs. Unlike single-target biologics (e.g., anti-IL-6 receptor monoclonal antibodies) and small-molecule kinase inhibitors (e.g., Janus kinase/STAT blockers), BAC simultaneously modulates IL-6/STAT3 signaling and activates ACE2. This dual mechanism makes BAC suitable for treating a wide range of comorbidities such as RA and cardiovascular diseases.

In summary, although the cardioprotective and anti-inflammatory effects of BAC are well known, systematic investigations into its multitarget mechanisms remain limited. Future studies should explore the relationships between BAC’s physiological activities and investigate its multipathway–multitarget synergistic mechanisms. Furthermore, researchers should incorporate network pharmacology analyses and perform systematic analyses from a holistic perspective. To enhance translational relevance, additional *in vitro* and *in vivo* experiments should be conducted to validate BAC’s interactions with IL-6, STAT3, and Akt1.

## 8 Future perspectives and unresolved challenges

Although BAC has considerable therapeutic potential, several challenges hinder its clinical application. First, sustained-release formulations must be developed to address the metabolite’s short half-life and improve treatment adherence in patients with chronic diseases such as arthritis. Second, leveraging the ACE2 agonist activity of BAC in precision medicine represents a promising area of research. For instance, stratifying patients with heart failure by biomarkers may help predict each patient’s response to BAC’s dual effects: cardioprotective (through STAT3 inhibition) and vasodilatory (through the ACE2/angiotensin one to seven pathway) effects. Finally, the synergistic effects of BAC with other drugs should be systematically evaluated, particularly in comparison of BAC with mainstream drugs such as IL-6 inhibitors for arthritis or SGLT2 inhibitors for heart failure, to determine whether BAC can improve treatment efficacy or reduce adverse reactions. Addressing these research gaps through interdisciplinary collaboration will expedite the clinical translation of BAC.

## Glossary

BAC: benzoylaconine
IL-6: interleukin-6
MAPK: mitogen-activated protein kinase
PI3K: Phosphatidylinositol 3-kinase
Akt: protein kinase B
STAT3: signal transducer and activator of transcription 3
MDA: monoester diterpenoid alkaloid
TCM: traditional Chinese medicine
AC: aconitine
PPI: protein–protein interaction
GO: gene ontology
KEGG: Kyoto Encyclopedia of Genes and Genomes
DO: disease ontology
MA: mesaconitine
HA: hypaconitine
ACN: aconine
MI: myocardial infarction
DDA: diester diterpenoid alkaloid
P-gp: P-glycoprotein
ROS: reactive oxygen species
BCRP: breast cancer resistance protein
MRP2: multidrug resistance–associated protein 2
ATP: adenosine triphosphate
ACE2: angiotensin-converting enzyme 2
RA: rheumatoid arthritis
LPS: lipopolysaccharide
YCZFD: Yinchenzhufu decoction
IL: interleukin
PGE2: prostaglandin 2
TNF-α: tumor necrosis factor α
HIF-α: hypoxia-inducible factor α
VEGF: vascular endothelial growth factor
TLR: toll-like receptor
CLF: cholestatic liver fibrosis
PDGFRβ: platelet-derived growth factor receptor-β.
