# Supplementary material for: Pharmacological effects, molecular mechanisms, and pharmacokinetics of benzoylaconine: a systematic review
Source: Front Pharmacol. 2025 Aug 5;16:1571153. doi: 10.3389/fphar.2025.1571153 (PMC12392121; doi:10.3389/fphar.2025.1571153)
Supplement: Supplementary file 1 [file Supplementaryfile1.docx]

**Appendix 1. Composition of polyherbal preparations and taxonomic validation**

**Polyherbal preparations**

**1.Yougui-Wan (YGW)**

YGW consists of ten herbs: Aconiti Lateralis Radix Praeparata (*Aconitum carmichaelii* Debx.), Cinnamomi Cortex (*Cinnamomum cassia* Presl.), Cervi Cornus Colla (*Cervus nippon* Temminck or *Cervus elaphus* L.), Rehmanniae Radix Praeparata (*Rehmannia glutinosa* Libosch.), Lycii Fructus (*Lycium barbarum* L.), Dioscoreae Rhizoma (*Dioscorea opposita* Thunb.), Corni Fructus (*Cornus officinalis* Sieb. et Zucc.), Cuscutae Semen (*Cuscuta chinensis* Lam.), Eucommiae Cortex (*Eucommia ulmoides* Oliv.), and Angelicae Sinensis Radix (*Angelica sinensis* (Oliv.) Diels). The herbal ratio is 2:2:4:8:4:4:3:4:4:3, respectively. The mixture was extracted with 50% alcohol by ultrasonication and concentrated to a final concentration of 4 g/ml.

**2.You-Gui-Yin (YGY)**

YGY consists of eight herbs: Aconiti Lateralis Radix Praeparata (*Aconitum carmichaelii* Debx.), Cinnamomi Cortex (*Cinnamomum cassia* Presl.), Rehmanniae Radix Praeparata (*Rehmannia glutinosa* Libosch.), Eucommiae Cortex (*Eucommia ulmoides* Oliv.), Corni Fructus (*Cornus officinalis* Sieb. et Zucc.), Dioscoreae Rhizoma (*Dioscorea opposita* Thunb.), Lycii Fructus (*Lycium barbarum* L.), and Glycyrrhizae Radix et Rhizoma (*Glycyrrhiza uralensis* Fisch.). The traditional dose ratio is 2:3:3:3:1:2:2:1, respectively.

1. **Sini-Tang (SND)**

SND consists of three herbs: Aconiti Lateralis Radix Praeparata (*Aconitum carmichaelii* Debx.), Glycyrrhizae Radix et Rhizoma Praeparata (*Glycyrrhiza uralensis* Fisch.), and Zingiberis Rhizoma (*Zingiber officinale* Rosc.).

1. **Shenfu-Tang (SHEN-FU)**

SHEN-FU mainly consists of two herbs: Ginseng Radix et Rhizoma Rubra (*Panax ginseng* C.A. Mey.) and Aconiti Lateralis Radix Praeparata (*Aconitum carmichaelii* Debx.).

1. **Fuzi-Tang (FZD)**

FZD consists of five herbs: Aconiti Lateralis Radix Praeparata (*Aconitum carmichaelii* Debx.), Poria (*Poria cocos* (Schw.) Wolf), Codonopsis Radix (*Codonopsis pilosula* (Franch.) Nannf.), Atractylodis Macrocephalae Rhizoma (*Atractylodes macrocephala* Koidz.), and Paeoniae Radix Alba (*Paeonia lactiflora* Pall.).

**6.Mahuang–Fuzi–Xixin-Tang (MFXT)**

MFXT consists of three herbs: Ephedrae Herba (*Ephedra sinica* Stapf), Aconiti Lateralis Radix Praeparata (*Aconitum carmichaelii* Debx.), and Asari Radix et Rhizoma (*Asarum sieboldii* Miq.).

**7.Yinchenzhufu decoction (YCZFD)**

YCZFD consists of six herbs: Artemisiae Scopariae Herba (*Artemisia capillaris* Thunb.), Atractylodis Macrocephalae Rhizoma (*Atractylodes macrocephala* Koidz.), Aconiti Lateralis Radix Praeparaia (*Aconitum carmichaelii* Debx.), Zingiberis Rhizoma (*Zingiber offificinale* Rosc.), Glycyrrhizae Radix et Rhizoma Praeparata (*Glycyrrhiza uralensis* Fisch.), and Cinnamomi Cortex (*Cinnamomum cassia* Presl.).

**8.Dahuang Fuzi Decoction (DFD)**

DFD consists of three herbs: Rhei Radix et Rhizoma (*Rheum palmatum* L. or *Rheum officinale* Baill.), Aconiti Lateralis Radix Praeparata (*Aconitum carmichaelii* Debx.), and Asari Radix et Rhizoma (*Asarum heterotropoides* Fr. Schmidt or *Asarum sieboldii* Miq.).

**Taxonomic Validation of Medicinal Plants**

**Fuzi**, Aconitum carmichaelii Debx. [Ranunculaceae; Aconiti Lateralis Radix Praeparata]

**Verify:** [MPNS](https://mpns.science.kew.org/mpns-portal/plantDetail?plantId=2618501&query=Aconitum+carmichaelii+Debx&filter=&fuzzy=false&nameType=latin&dbs=wcsCmp)

**Ganjiang**, Zingiber officinale Roscoe [Zingiberaceae; Zingiberis Rhizoma]

**Verify:** [MPNS](https://mpns.science.kew.org/mpns-portal/searchName?searchTerm=Zingiber+officinale%C2%A0Roscoe&nameType=latin)

**Chuanwu**, Aconitum carmichaelii Debx. [Ranunculaceae; Aconiti Radix]

**Verify:** [MPNS](https://mpns.science.kew.org/mpns-portal/searchName?searchTerm=Aconitum+carmichaelii%C2%A0Debx&nameType=latin)

**Ephedrae Herba (Mahuang)**, Ephedra sinica Stapf [Ephedraceae; Ephedrae Herba

**Verify:** [MPNS](https://mpns.science.kew.org/mpns-portal/searchName?searchTerm=Ephedra+sinica%C2%A0Stapf&nameType=latin)
